# The Week's Work

**Published:** 1911-04-01

**Authors:** 


### The Week's Work.

### A NOTE FOR HOSPITAL SECRETARIES.

In these days when so much is heard about hospital abuse, especially so far as the out-patient department is concerned, the action taken by the Seamen's Hospital Society with regard to the Dreadnought Hospital at Greenwich is deserving of attention, and, where possible, of emulation by other hospital authorities. In November last the Medical Superintendent of the Dreadnought issued a letter to the practitioners resident in the district round about the hospital, enclosing a set of cards which bore on the obverse, as numismatists say, the following detail: "The Dreadnought Hospital is for the treatment of seamen, accidents, and emergencies. A few landsmen, women, and children are received and treated as out-patients, but they must show evidence of being unable to afford a doctor's fee. The bearer ———————— is therefore required to obtain a doctor's signature on the back of this card certifying that he or she is a fit case for hospital treatment." On the reverse—once more to follow the numismatists—it bore a simple inscription: "I certify that the bearer is a fit and proper subject for hospital treatment. Signed, ————————. Date ————————." The issue of these cards is obviously in the interests alike of the hospital and of the medical practitioners in the neighbourhood. The Committee of the Dreadnought has taken a commendable step towards protecting the Greenwich doctors from unfair competition and the out-patient department from abuse. Already, we are informed, the innovation has been highly successful. Certainly those local practitioners whose opinion we have been able to obtain think that it is an improvement on pre-existing methods—or rather the want of them. There already exists at Greenwich a provident dispensary that is doing good work on a small scale, and the medical practitioners there have shown that they are cordially willing to co-operate with the hospital authorities in putting down abuse of the out-patient department. In the circumstances we shall watch with interest the reports of the hospital to see how the system works, and in the meantime we commend its adoption to other hospitals who may be able to plead with as much justice as the Dreadnought does that they cater for a special class of patients only. These institutions are in a far better position, it must be admitted, to cope with the problem of out-patient abuse than their fellow-hospitals; but even the latter may seriously consider the advisability of appealing in so direct, and we are inclined to think, so pertinent a fashion to the medical profession for support and co-operation.

### HOSPITAL BALCONIES.

One of the problems that the hospital constructor has to deal with is the proper relation of balcony to ward space, and the satisfactory disposal of his balconies. When it is desired to add new balconies to an already existing building, especially when the structure is old and of an obsolete type, the problem is even greater. We have in mind a London hospital that is contemplating such a reformative scheme, and we understand that the building committee has been much worried over the matter and has not as yet decided on a definite plan. When we turn to books on hospital architecture we find that the balcony is dismissed with comparatively scant attention. Some years ago Dr. Sarason pointed this out, and evolved a scheme for a terrace system of balconies which bids fair to supersede the present arrangement of overlapping whereby the lower balconies are shut out from a fair share of sunlight. At the time we drew attention to Dr. Sarason's paper and commented on its importance to hospital constructors. Since then no further progress appears to have been made in the building of balconies. In new buildings the Sarason's terrace system is of course easy to instal; in existing hospitals, where it is impossible to push the end wall back for a yard or more, its adoption is surrounded by so many difficulties that it is almost impracticable. Nevertheless it is always a system that should be considered, and especially in a climate so murky as ours its adoption should be carefully discussed when planning a new hospital. We understand that the system has been adopted both in America and on the Continent: in England it is yet so little known that we make no apology for once more drawing attention to the résumé of the original paper that appeared in The Hospital. It is certainly a system that may with some justification be styled revolutionary. How far it is likely to answer in actual practice remains to be seen when it has been tried fairly in a large hospital under conditions sufficiently adverse to test its merits to the full.

### HOSPITALS AND AMALGAMATION.

Co-operation is replacing unrestricted competition in the hospital world as everywhere else. The result is that few annual meetings take place without the question of the amalgamation of local institutions being raised. At Edinburgh a great scheme has been advocated publicly; at Bradford certain local difficulties have been put down to the want of it; at Bristol the sheriff and a member of the medical staff of the Children's Hospital introduced and supported, in general terms, the same principle. These are examples taken at haphazard, examples which could be added to without difficulty, and they establish the tendency to centralise. This tendency is seen in two ways, in the outcries against overlapping which affect the relations of one hospital to another, and which end in proposals for amalgamation, and in the architectural development of the hospital itself. That has not ended with the practical establishment of the pavilion system. The pavilion system seems rather to be the first step towards a grouping of hospital buildings on a common site, which is now beginning to be chosen outside the city. Casualty departments will be left to deal with accidents and emergencies generally, other cases will be dealt with at the hospital centre or city, away from the noise and smoke of busy streets. This, it will be admitted, is the tendency in the architectural development of hospitals to-day. It is instructive to find the same principle at work also in hospital management.

### THIS WEEK'S DRUG MARKET.

Business continues rather quiet, but has somewhat improved since last week. On the whole, prices are well maintained, but there is a notable exception in the case of quicksilver, the price of which has been considerably reduced. An advance in the value of glycerine is not altogether unexpected. Cascara sagrada is tending dearer, as also is jalap. Turpentine has advanced to an extremely high figure. High prices have been paid for asafœtida. Ipecacuanha is rather lower in value, and Damiana leaves have been sold at cheaper rates. There is no decline in the quotations for cod-liver oil, but the weather in the Norwegian fishing grounds has been more favourable, and there has been an improved catch. Up to date, however, the total yield of oil is not more than two-thirds of the yield at this time last year. The position in the opium market is practically unchanged. Oil of cloves has a slightly dearer tendency. Orange peel is considerably dearer. Camphor is unchanged.

